# Salvage Reirradiation Options for Locally Recurrent Prostate Cancer: A Systematic Review

**DOI:** 10.3389/fonc.2021.681448

**Published:** 2021-09-09

**Authors:** Jim Zhong, Finbar Slevin, Andrew F. Scarsbrook, Maria Serra, Ananya Choudhury, Peter J. Hoskin, Sarah Brown, Ann M. Henry

**Affiliations:** ^1^Department of Diagnostic and Interventional Radiology, Leeds Cancer Centre, St James’s University Hospital, Leeds Teaching Hospitals NHS Trust, Leeds, United Kingdom; ^2^Leeds Institute of Medical Research, University of Leeds, Leeds, United Kingdom; ^3^Department of Clinical Oncology, Leeds Cancer Centre, St James’s University Hospital, Leeds Teaching Hospitals NHS Trust, Leeds, United Kingdom; ^4^Department of Clinical Oncology, The Christie Hospital, Manchester, United Kingdom; ^5^The Institute of Cancer Sciences, University of Manchester, Manchester, United Kingdom; ^6^Department of Clinical Oncology, Mount Vernon Cancer Centre, Northwood, United Kingdom; ^7^Clinical Trials Research Unit, University of Leeds, Leeds, United Kingdom

**Keywords:** prostate cancer, local recurrence, reirradiation, salvage, brachytherapy, external beam radiotherapy (EBRT), stereotactic body radiotherapy (SBRT)

## Abstract

**Background:**

Reirradiation using brachytherapy (BT) and external beam radiation therapy (EBRT) are salvage strategies with locally radiorecurrent prostate cancer. This systematic review describes the oncologic and toxicity outcomes for salvage BT and EBRT [including Stereotactic Body Radiation Therapy (SBRT)].

**Methods:**

An International Prospective Register of Systematic Reviews (PROSPERO) registered (#211875) study was conducted using Preferred Reporting Items for Systematic reviews and Meta-analyses (PRISMA) guidelines. EMBASE and MEDLINE databases were searched from inception to December 2020. For BT, both low dose rate (LDR) and high dose rate (HDR) BT techniques were included. Two authors independently assessed study quality using the 18-item Modified Delphi technique.

**Results:**

A total of 39 eligible studies comprising 1967 patients were included (28 BT and 11 SBRT). In 35 studies (90%), the design was single centre and/or retrospective and no randomised prospective studies were found. Twelve BT studies used LDR only, 11 HDR only, 4 LDR or HDR and 1 pulsed-dose rate only. All EBRT studies used SBRT exclusively, four with Cyberknife alone and 7 using both Cyberknife and conventional linear accelerator treatments. Median (range) modified Delphi quality score was 15 (6-18). Median (range) follow-up was 47.5 months (13-108) (BT) and 25.4 months (21-44) (SBRT). For the LDR-BT studies, the median (range) 2-year and 5-year bRFS rates were 71% (48-89.5) and 52.5% (20-79). For the HDR-BT studies, the median (range) 2-year and 5-year bRFS rates were 74% (63-89) and 51% (45-65). For the SBRT studies, the median (range) 2-year bRFS for the SBRT group was 54.9% (40-80). Mean (range) acute and late grade≥3 GU toxicity rates for LDR-BT/HDR-BT/SBRT were 7.4%(0-14)/2%(0-14)/2.7%(0-8.7) and 13.6%(0-30)/7.9%(0-21.3%)/2.7%(0-8%). Mean (range) acute and late grade≥3 GI toxicity rates for LDR-BT/HDR-BT/SBRT were 6.5%(0-19)/0%/0.5%(0-4%) and 6.4%(0-20)/0.1%(0-0.9)/0.2%(0-1.5). One third of studies included Patient Reported Outcome Measures (PROMs).

**Conclusions:**

Salvage reirradiation of radiorecurrent prostate cancer using HDR-BT or SBRT provides similar biochemical control and acceptable late toxicity. Salvage LDR-BT is associated with higher late GU/GI toxicity. Challenges exist in comparing BT and SBRT from inconsistencies in reporting with missing data, and prospective randomised trials are needed.

## Introduction

Prostate cancer is the most common male cancer accounting for over 1.2 million new cases per year with >350,000 deaths (3.8% of all male cancer deaths) (1). Radiation therapy (RT) is a curative treatment option for localised prostate cancer and can be offered to patients from all risk groups (2). Despite advances in diagnostic imaging, RT delivery techniques and dose-escalated radiation, biochemical progression remains common and occurs in 15–57% of patients with localised disease (3–5).

Multiple salvage options are available for locally recurrent non-metastatic disease including prostatectomy, reirradiation [with brachytherapy (BT) or external beam radiotherapy (EBRT)] and other focal therapies such as high-intensity focused ultrasound (HIFU) and cryotherapy. However, there is limited evidence to support the effectiveness of salvage therapies with concerns regarding the potential for significant toxicity that may impact the long-term quality of life of patients. Due to uncertainty regarding benefits and risks of harm only 15-20%of patients with locally recurrent prostate cancer undergo salvage therapy according to the Cancer of the Prostate Strategic Urologic Research Endeavor (CaPSURE) registry (6).

BT has been preferred for reirradiation as it offers delivery of highly conformal high dose radiation with a steep dose gradient and rapid fall off which minimises dose to surrounding organs at risk (7). Disadvantages of BT include its invasive nature and the need for a specialist multi-disciplinary team not available in all radiation centres. Previously, EBRT techniques have been associated with high rates of severe late toxicities and poor local control (8). Stereotactic body radiation therapy (SBRT) involves delivery of a high dose of external beam radiation to a highly conformal target volume with a steep dose gradient in a small number of fractions and is now under investigation for locally recurrent prostate cancer. Advantages of this approach are that it is non-invasive and has the potential to be delivered in more radiation centres than BT (9).

This systematic review collates the most up-to-date evidence for reirradiation of locally recurrent prostate cancer. Two previous systematic reviews which compared all salvage therapies found higher biochemical control rates for BT and EBRT compared to surgical and other non-surgical local therapies [high intensity focused ultrasound (HIFU) and cryotherapy] along with potentially lower genitourinary (GU) toxicity (10, 11). The justification for this systematic review is that the identification of the reirradiation modality that offers optimum prostate cancer control and minimal toxicity is important to enable patients to make informed decisions and potentially improve outcomes in patients with radiorecurrent prostate cancer. In addition, the evidence base for salvage BT and SBRT continues to expand with a number of new publications in the past 1-2 years.

## Methods and Materials

An International Prospective Register of Systematic Reviews registered (#211875) systematic review was conducted.

### Study Design

The study followed the Preferred Reporting Items for Systematic Reviews and Meta-analysis (PRISMA) guidelines (12).

Studies were identified by searching the Cochrane library, EMBASE and MEDLINE electronic databases from inception to 14^th^ December 2020.

The search strategy is documented in **Supplementary Material 1** and the combination of subject headings and keywords included: ‘recurrent or radiorecurrent prostate cancer’, ‘reirradiation’ or ‘re-irradiation’, ‘salvage radiotherapy’, ‘brachytherapy’, ‘external beam radiotherapy’, ‘stereotactic body radiation therapy’, ‘stereotactic ablative radiotherapy’, ‘radiosurgery’.

### Data Extraction

Two authors (JZ and FS) independently reviewed the abstracts and assessed the quality of each study using an 18-item Modified Delphi technique, which has been previously validated for case series (13). Discordance between reviewers were resolved following arbitration by a third reviewer (AH).

### Data Selection

Eligible studies included patients treated with primary EBRT, BT or combination EBRT/BT and salvage therapy for local recurrence with either EBRT or BT. For BT techniques, studies of high-dose rate brachytherapy (HDR-BT), low-dose rate brachytherapy (LDR-BT) and pulse-dose rate brachytherapy (PDR-BT) were included.

Studies that predominantly included patients who had primary treatment with radical prostatectomy, cryotherapy or HIFU were not included in this review as the focus was to collate and present the most up-to-date evidence concerning reirradiation specifically.

Studies with fewer than 20 patients were excluded, along with editorials, letters, abstracts, case reports, conference proceedings and studies not written in English. Where studies had evaluated the same patient cohort as another publication, only the most recent publication was used for analysis unless distinct treatment outcomes or toxicity were described.

## Extracted Variables

Extracted data included the first author and country in which the study took place, study type (prospective or retrospective), single/multi-centre status, number of patients, primary disease characteristics, primary treatment modalities, interval between original treatment and salvage treatment, patient age at salvage, pre-salvage prostate specific antigen (PSA), diagnostic imaging prior to salvage treatment, histological confirmation of local recurrence and percentage of biopsy-proven recurrences in study cohort, whole-gland salvage treatment versus focal salvage treatment, type of salvage radiotherapy (HDR-BT, LDR-BT, PDR-BT or EBRT), salvage dose fractionation schedule, percent of patients who received androgen deprivation therapy (ADT) with their salvage treatment, duration of follow up post salvage therapy, treatment outcomes [biochemical control (BC), biochemical recurrence free survival (bRFS), metastasis free survival (MFS), relapse free survival (RFS), cancer specific survival (CSS), overall survival (OS)] and grade 1-4 GU and gastrointestinal (GI) toxicity as classified by the Common Terminology Criteria for Adverse Events (CTCAE) (14) or Radiation Therapy Oncology Group (RTOG) score (15). Use of any patient recorded outcome measure (PROM) in the study was also collated including the type of tool used. Median (range) values calculated for all collected variables except toxicity rates where mean (range) used to account for the studies which report no toxicity.

## Results

**Figure 1:**
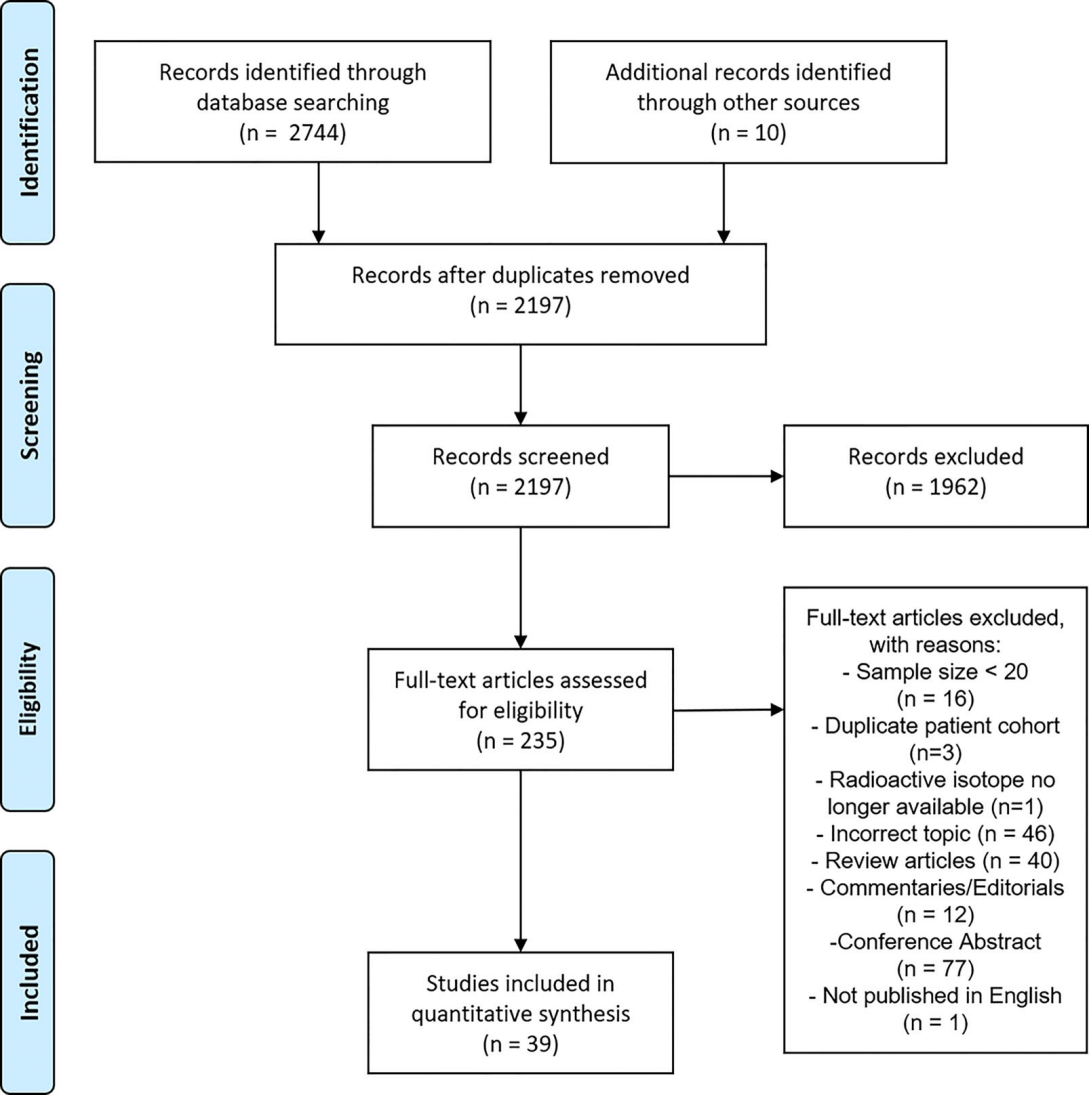
PRISMA flow chart of literature search.

From the initial identification of 2744 articles, a total of 39 studies were included in the final analysis. A PRISMA flowchart of the systematic review is presented in **Figure 1**. The last electronic literature search was performed on 14^th^ December 2020.

The quality assessment tool (modified Delphi 18-item checklist) scores for all included studies are shown in **Supplementary Table 1**. The summary of these results is shown in **Table 1**. The median modified Delphi score was 15 out of 18 (83.3%) (range 6-18).

**Table 1:** Summary findings from the Modified Delphi checklist for quality assessment applied to all included studies (n=39).

Criterion	Studies, n (%)	
	Yes	No
**Study Objective**		
1. Is the hypothesis/aim/objective of the study clearly stated in the abstract, introduction, or methods section?	38 (97.4)	1 (2.6)
**Study population**		
2. Are the characteristics of the participants included in the study described?	100 (100)	0 (0)
3. Were the cases collected in more than 1 Centre?	8 (20.5)	31 (79.5)
4. Are the eligibility criteria (inclusion and exclusion criteria) to entry the study explicit and appropriate?	33 (84.6)	6 (15.4)
5. Were the participants recruited consecutively?	26 (66.7)	13 (33.3)
6. Did participants enter the study at a similar point in the disease?	35 (89.7)	4 (10.3)
**Intervention and co-intervention**		
7. Was the intervention clearly described in the study?	37 (94.9)	2 (5.1)
8. Were additional interventions (co-interventions) clearly reported in the study?	35 (89.7)	4 (10.3)
**Outcome measures**		
9. Are the outcome measures clearly defined in the introduction or methods section?	38 (97.4)	1 (2.6)
10. Were relevant outcomes appropriately measured with objective/or subjective methods?	38 (97.4)	1 (2.6)
11. Were outcomes measured before and after intervention?	35 (89.7)	4 (10.3)
**Statistical analysis**		
12. Were the statistical tests used to assess the relevant outcomes appropriate?	38 (97.4)	1 (2.6)
**Results and conclusions**		
13. Was the length of follow-up reported?	38 (97.4)	1 (2.6)
14. Was the loss of follow-up reported?	23 (59.0)	16 (41.0)
15. Does the study provide estimates of the random variability in the data analysis of relevant outcomes?	15 (38.5)	24 (61.5)
16. Are adverse events reported?	38 (97.4)	1 (2.6)
17. Are the conclusions of the study supported by results?	38 (97.4)	1 (2.6)
**Competing interest and source of support**		
18. Are both competing interest and source of support for the study reported?	23 (59.0)	16 (41.0)
**Median Modified Delphi score = 15 out of 18 (83.3%) (range 6-18)**		

### Treatment Details

A summary of patient, disease and treatment characteristics at the time of primary treatment and salvage treatments for BT and EBRT studies is shown in **Tables 2**–**5** respectively. Salvage treatment characteristics for BT and EBRT are shown in **Tables 6**, **7** respectively.

**Table 2:** Primary disease and treatment characteristics for brachytherapy studies.

First author (country)	Year	Salvage BT type	Design	Pts (*n*)	PSA (ng/mL)(range)	ISUP	GS	% GS (≤7)	% GS (≥8)	T stage	% T stage (≤T2a)	% T stage (≥T2b)	Risk Class	Primary treatment
B Lee (USA)	2007	HDR	R	21	NR	1	6	100	0	T2c	48	52	NR	EBRT, BT, protonTx
Lyczek (Poland)	2009	HDR	R	115	13 (2.34-64.5)	NR	NR	NR	NR	T2	58	42	NR	RP+EBRT, EBRT, BT, EBRT+BT
Chen (USA)	2013	HDR	R	52	9.3 (1.2-58)	1	6	87	13	T2	NR	NR	NR	EBRT, BT, EBRT+BT, PBT
Kukielka (Poland)	2014	HDR	R	25	16.3 (6.37-64)	1	<6	88	4	T2c	48	52	Intermediate	EBRT
*Yamada (USA)	2014	HDR	P	42	NR	NR	NR	NR	NR	NR	NR	NR	NR	EBRT
Jiang (Germany)	2016	HDR	R	29	NR	NR	NR	NR	NR	NR	NR	NR	High	EBRT, EBRT+BT
Lacy (USA)	2016	HDR	R	21	NR	NR	NR	NR	NR	NR	NR	NR	Low	BT, EBRT+BT
Wojcieszek (Poland)	2016	HDR	R	83	13.7	1	6	80	4	T2	NR	NR	Intermediate	EBRT, EBRT+BT
Lopez (Spain)	2019	HDR	R	75	8.9 (3.5-42.1)	1	6	75	20	NR	NR	NR	Intermediate	EBRT, BT
	2019	LDR	R	44	14.2 (3.2-167)	1	6	87	11	NR	NR	NR	Intermediate	EBRT
Chitmanee (UK)	2020	HDR	P	50	<10 (46%)	2/3	7	90	10	T2	72	28	Intermediate	EBRT, BT
Slevin (UK)	2020	HDR	R	43	10.5 (3.4-178)	1	6	90	10	T2	73%	27%	Intermediate	EBRT, BT
van Son (Netherlands)	2020	HDR	P	50	13 (2.1-140)	1	6	82	12	T2a	72	28	NR	EBRT, BT
Kollmeier (USA)	2017	HDR/LDR	R	98	≤10 (74%)	2	7	92	8	T2b	68	32	NR	EBRT, BT, EBRT+BT
Baumann (USA)	2017	HDR/LDR	R	33	8.4 (3.8-68.7)	NR	7	79	21	T2	55	45	High	EBRT
Henriquez (Spain)	2014	HDR/LDR	R	56	10.7 (4-121)	1	6	95	5	T2	87	13	Intermediate	EBRT, BT
Grado (USA)	1999	LDR	R	49	26.4 (2.3-95.8)	NR	7 to 10	NR	NR	T2b	NR	NR	NR	EBRT, BT, RP
Koutrouvelis (USA)	2003	LDR	R	31	<10 (32%)	NR	6	77	23	T2b/T3a	32%	68%	NR	BT
Nguyen (USA)	2007	LDR	P	25	7.4 (4.2-18.4)	1	6	100	0	T1c	NR	NR	NR	EBRT, EBRT+BT
HK Lee (USA)	2008	LDR	R	21	NR	NR	NR	NR	NR	NR	NR	NR	NR	EBRT
Aaronson (USA)	2009	LDR	R	24	9.9 (3.2-69)	3	7	71	12	T1c	NR	NR	NR	EBRT
Burri (USA)	2010	LDR	R	37	10.9 (4.4-81)	NR	6	73	11	NR	19	16	Intermediate	EBRT, BT
Moman (Netherlands)	2010	LDR	R	31	24.3	NR	7	84	6.5	T2	NR	NR	NR	EBRT, BT
Peters (Netherlands)	2014	LDR	R	20	12.9 (5.4-51)	1	6	90	10	T3	NR	NR	High	EBRT, BT
Vargas (USA)	2014	LDR	R	69	<10 (62%)	1	6	80.3	19.7	T2	NR	NR	NR	EBRT
Peters (Netherlands)	2016	LDR	R	62	16.6 (2.6-66.9)	2/3	7	95	5	T2	66	34	NR	EBRT, BT
Crook (Canada)	2019	LDR	P	92	NR	NR	7	100	0	NR	NR	NR	low/intermediate	EBRT
Smith (USA)	2020	LDR	P	108	9.15 (1.7-116)	1	6	54	10	T2	67	5	Intermediate	EBRT
Schonle (Germany)	2020	PDR	R	82	9 (0.9-170)	2/3	7	74	11	NR	NR	NR	Intermediate	EBRT, BT, RP

BT, brachytherapy; HDR, high dose rate; LDR, low dose rate; R, retrospective; P, prospective; Pts, patients, n, nnumber; PSA, prostate specific antigen; NR, not recorded; GS, Gleason score; EBRT, external beam radiotherapy; PBT, proton beam treatment; RP, radical prostatectomy.

For PSA, ISUP and GS, the median scores are presented.

*Yamada (USA) study cohort included in further publication Kollmeier (USA) however specific treatment characteristics and toxicity were not covered in later paper.

**Table 3:** Pre-salvage therapy disease and treatment characteristics for brachytherapy studies.

First author (country)	Year	Salvage BT type	TRS (*mo*)(range)	BCR definition	Age (years)(range)	PSA (ng/mL)(range)	ISUP	GS	% GS (≤7)	% GS (≥8)	Imaging for relapse	Biopsy
B Lee (USA)	2007	HDR	63.6 (24-125)	NR	68 (58-81)	5.9 (1.4-9.5)	NR	NR	52	38	MRI	Yes (100%)
Lyczek (Poland)	2009	HDR	49.5	NR	70 (52-82)	NR	1	6	71	12	NR	No
Chen (USA)	2013	HDR	51.6 (10.8-135.6	Phoenix	67.5 (53.9-81.4)	5 (0.4-26.3)	NR	8	48	52	CT	Yes (100%)
Kukielka (Poland)	2014	HDR	NR	Phoenix	71 (62-83)	2.8 (1.04-25.3)	2/3	7	60	20	MRI	Yes (100%)
*Yamada (USA)	2014	HDR	73	Phoenix	72	3.54	NR	7	67	33	MRI, BS	Yes (100%)
Jiang (Germany)	2016	HDR	NR	Phoenix	75.5 (±5.8)	4.05 (2.1-18.6)	NR	NR	NR	NR	C-PET	No
Lacy (USA)	2016	HDR	45 (4-287)	Phoenix	59 (44-72)	6.3 (1-19.1)	NR	NR	NR	NR	CT, BS	Yes (14%)
Wojcieszek (Poland)	2016	HDR	67 (22-124)	NR	70 (57-81)	3.1 (0.1-19.9)	NR	7	46	7	MRI, BS	Yes (100%)
Lopez (Spain)	2019	HDR	> 30	ASTRO/ Phoenix	62.1 (4-75)	4.1 (1.5-16.7)	NR	8 to 10	48	44	CT, MRI, C-PET	Yes (100%)
	2019	LDR	> 30	ASTRO/ Phoenix	60.4 (47-71)	3.6 (1.02-11)	2/3	7	59	9	CT, MRI, C-PET	Yes (100%)
Chitmanee (UK)	2020	HDR	< 5 years	Phoenix	70 (57-82)	<10 (94%)	2/3	7	54	36	MRI, C-PET	Yes (100%)
Slevin (UK)	2020	HDR	70	Phoenix	70 (62-81)	3.1 (1.1-7.5)	2	7	70	30	MRI, PET	Yes (100%)
van Son (Netherlands)	2020	HDR	101 (25-228)	Phoenix	71 (59-83)	5 (0.9-39)	2	7	74	20	PSMA-PET, MRI	Yes (100%)
Kollmeier (USA)	2017	HDR/LDR	72 (12-172)	Phoenix	73.5 (56-88)	3.7 (0-59)	2	7	61	39	CT, MRI, BS	Yes (100%)
Baumann (USA)	2017	HDR/LDR	56.1 (18-118)	Phoenix	75 (57-85)	5 (2-26)	NR	7	55	36	CT, MRI, BS	Yes (100%)
Henriquez (Spain)	2014	HDR/LDR	NR	Phoenix	65 (60-80)	3.7 (1.1-30)	2/3	7	41	14	MRI	Yes (100%)
Grado (USA)	1999	LDR	NR	2 PSA rises>nadir	73.3 (52.9-86.9)	5.6 (1.5-79.1)	NR	NR	NR	NR	CT	Yes (100%)
Koutrouvelis (USA)	2003	LDR	30	nPSA+1.5	65 (51-79)	NR	NR	6	NR	NR	NR	Yes (100%)
Nguyen (USA)	2007	LDR	62.4 (30-153)	ASTRO	65	5.5 (1.4-11.6)	NR	NR	NR	NR	MRI	Yes (100%)
HK Lee (USA)	2008	LDR	85 (±30.1)	Phoenix	72 (±4.8)	3.8	NR	7	NR	NR	NR	Yes (100%)
Aaronson (USA)	2009	LDR	49 (26-109)	Phoenix	66 (54-88)	3.41 (0.3-10)	NR	NR	NR	NR	MRI	Yes (100%)
Burri (USA)	2010	LDR	62 (26-171)	Phoenix	70.2 (51-79)	5.6 (1.7-35)	NR	8	65	32	CT	Yes (100%)
Moman (Netherlands)	2010	LDR	60	ASTRO/ Phoenix	69.3	11.4	NR	8	70.1	12.9	NR	Yes (100%)
Peters (Netherlands)	2014	LDR	79 (42-144)	Phoenix	69 (59-78)	4.7 (0.3-14)	NR	7	65	35	MRI, CT/BS, C-PET	Yes (100%)
Vargas (USA)	2014	LDR	90	Phoenix	72.5 (55-88)	NR	NR	7	73.2	26.8	CT, BS	Yes (100%)
Peters (Netherlands)	2016	LDR	67 (±32)	Phoenix	69 (±5.3)	8.6 (0.1-92.6)	NR	NR	NR	NR	MRI, PET, BS	Yes (100%)
Crook (Canada)	2019	LDR	85 (39-199)	Phoenix	70 (55-82)	4.1 (0.4-9.7)	NR	NR	NR	NR	MRI	Yes (100%)
Smith (USA)	2020	LDR	70 (10-235)	Phoenix	70 (51-87)	5.3 (0.1-38.4)	3	7	65	32	MRI	Yes (100%)
Schonle (Germany)	2020	PDR	87.5 (19-255)	Phoenix	69.9 (51-83)	5.07 (0.28-51)	2/3	7	59	24	MRI	NR

BT, brachytherapy; HDR, high dose rate; LDR, low dose rate; PSA, prostate specific antigen; NR, not recorded; GS, Gleason score; TRS, median time from primary treatment to salvage therapy; mo, months; BCR, biochemical recurrence; ASTRO, American Society for Radiation Oncology; MRI, magnetic resonance imaging; NS, bone scan; CT, computed tomography; US, ultrasound; C-PET, Choline positron emission tomography; PSMA, prostate specific membrane antigen.

For TRS, age, PSA, ISUP and GS, the median scores are presented.

*Yamada (USA) study cohort included in further publication Kollmeier (USA) however specific treatment characteristics and toxicity were not covered in later paper.

**Table 4:** Primary disease and treatment characteristics for EBRT studies.

First author/ country	Year	Design	Pts (*n*)		PSA (range) (ng/mL)	ISUP	GS		% GS (≤7)	% GS (≥8)	T stage	% T stage (≤T2a)	% T stage (≥T2b)	Risk Class	Primary treatment
Leroy (France)	2017	R	23		10.38 (2.34-57)	2/3	7		82.5	4.3	T2	65.2	30.4	NR	EBRT, BT
Fuller (USA)	2020	P	50		NR	NR	NR		NR	NR	NR	NR	NR	NR	EBRT, BT, RP
Jereczek-Fossa (Italy)	2018	R	64		11.4 (0.5-228.5)	2/3	7		NR	NR	NR	NR	NR	NR	EBRT, BT
Loi (Italy)	2018	R	50		10 (3.1-160)	NR	NR		70	30	NR	NR	NR	High	EBRT, RP+EBRT
D'Agostino (Italy)	2019	R	23		NR	NR	NR		NR	NR	NR	NR	NR	Intermediate	RP+EBRT, EBRT
Pasquier (France)	2019	R	100		10.2 (2.3-120)	1	6		93	7	NR	NR	NR	Intermediate	EBRT
Scher (France)	2019	R	42		10.1 (3-120)	2/3	7		82	18	NR	NR	NR	Intermediate	EBRT, RP+EBRT
Cuccia (Italy)	2020	R	24		NR	3	7		79	21	NR	NR	NR	Intermediate	EBRT, BT
Matrone (Italy)	2020	R	44		8.7 (2.6-46)	2/3	7a		NR	NR	NR	NR	NR	High	EBRT
Caroli (Italy)	2020	R	38		NR	2	7		100	0	T3	42.1	57.9	NR	EBRT, RP+EBRT
Bergamin (Australia)	2020	P	25		13 (4.1-97)	2	7		72	28	T2a	80	20	Intermediate	EBRT, BT

BT, brachytherapy; HDR, high dose rate; LDR, low dose rate; R, retrospective; P, prospective; Pts, patients; n, number; PSA, prostate specific antigen; NR, not recorded; GS, Gleason score; EBRT, external beam radiotherapy; PBT, proton beam treatment; RP, radical prostatectomy.

For PSA, ISUP and GS, the median scores are presented.

**Table 5:** Pre-salvage therapy disease and treatment characteristics for EBRT studies.

First author/ country	Year	Design	TRS (*mo*)(range)	BCR definition	Age (years)(range)	PSA (ng/mL)(range)	ISUP	GS	% GS (≤7)	% GS (≥8)	Imaging for relapse	Biopsy
Leroy (France)	2017	R	65 (28-150)	Phoenix	70 (58-82)	2.5 (0-11.7)	NR	NR	NR	NR	C-PET, MRI	Yes (83%)
Fuller (USA)	2020	P	98 (31-241)	Phoenix	74 (50-89)	3.97 (0.1-48.2)	3	7	64	36	MRI	Yes (100%)
Jereczek-Fossa (Italy)	2018	R	99.7 (23-208)	Phoenix	73.2 (52.6-81.7)	3.89 (0.17-51.8)	2/3	7	NR	NR	C-PET, MRI, CT	Yes (44%)
Loi (Italy)	2018	R	76 (9-205)	Phoenix	76 (62-86)	2.6 (1-30)	NR	NR	NR	NR	C-PET, MRI	NR
D'Agostino (Italy)	2019	R	90 (26-138)	NR	78 (69-85)	3.2 (1.2-13.5)	NR	NR	NR	NR	C-PET	No
Pasquier (France)	2019	R	90 (24-216)	Phoenix	71.2 (56-86)	4.3 (2.0-38.3)	3	7	66	34	C-PET, MRI	Yes (100%)
Scher (France)	2019	R	82.5 (29-207)	Phoenix	64 (49-77)	3.1 (0.01-23.7)	NR	NR	NR	NR	C-PET, MRI	Yes (80%)
Cuccia (Italy)	2020	R	69 (29-141)	Phoenix	75 (65-89)	1.79 (0.18-10)	NR	NR	NR	NR	C-PET/ PSMA-PET, MRI	No
Matrone (Italy)	2020	R	60 (16.9-615.5)	Phoenix	76 (56-89)	2.6 (2-7.68)	1	6	NR	NR	MRI, C-PET	Yes (11%)
Caroli (Italy)	2020	R	NR	Phoenix	75 (71-80)	1.1 (0.82-2.59)	NR	NR	NR	NR	PSMA-PET	NR
Bergamin (Australia)	2020	P	99.6 (54-163.2)	Phoenix	72 (62-83)	4.1 (1.1-16.6)	NR	NR	NR	NR	PSMA-PET	Yes (100%)

BT, brachytherapy; HDR, high dose rate; LDR, low dose rate; PSA, prostate specific antigen; NR, not recorded; GS, Gleason score; TRS, median time from primary treatment to salvage therapy; mo, months; BCR, biochemical recurrence; ASTRO, American Society for Radiation Oncology; MRI, magnetic resonance imaging; NS, bone scan; CT, computed tomography; US, ultrasound; C-PET, Choline positron emission tomography; PSMA , prostate specific membrane antigen.

For TRS, age, PSA, ISUP and GS, the median scores are presented.

**Table 6:** Salvage therapy details for BT studies.

First author (country)	Year	Single-centre (1) or Multi-centre (2)	Patients (n)	BT Technique	Radiation Source	Focal or Whole-gland	Dose (total dose (Gy)/ dose per fraction/ number of fractions)	Duration of treatment	Adjuvant ADT	Follow-up (mo) (range)	BC (%)	Oncologic outcomes
B Lee (USA)	2007	1	21	HDR	Ir-192	Whole	36/6/6	7 days	No	18.7	90.8	2-yr bRFS 89%
Lyczek (Poland)	2009	1	115	HDR	Ir-192	Whole	30 / 10 / 3	9 weeks	NR	NR	46 (PSA<6) vs 18 (PSA>6)	OS 86% (PSA<6) vs 48% (PSA>6)
Chen (USA)	2013	1	52	HDR	Ir-192	Whole	36 / 6 / 6	10 days	NR	59.6 (5.9-154.7)	55.7	5-yr bRFS 51%, 5-yr OS 92%
Kukielka (Poland)	2014	1	25	HDR with interstitial hyperthermia	Ir-192	Whole	37924	63 days	Yes (12%)	13 (4-48)	NR	2-yr bRFS 74%
*Yamada (USA)	2014	1	42	HDR	Ir-192	Whole	32 / 8 / 4	30 hours	Yes (43%)	36 (2-66)	68.5	5-yr OS 90.3%
Jiang (Germany)	2016	1	29	HDR	Ir-192	Whole	30 / 10 / 3	3 weeks	Yes (34.5%)	73 (61-140)	45	5-yr bRFS 45%, 5-yr OS 95.5%
Lacy (USA)	2016	1	21	HDR	Ir-192	Whole	108-144 Gy	–	Yes (14.3%)	61 (10-149)	47.6	NR
Wojcieszek (Poland)	2016	1	83	HDR	Ir-192	Whole	30 / 10 / 3	28-30 days	Yes (53%)	41 (11-76)	67	5-yr CSS 87%
Lopez (Spain)	2019	2	75	HDR	Ir-192	Whole	32 / 7-10 / 2-4	–	Yes (45%)	52	67.5	5-yr bRFS 65%
			44	LDR	NR	Whole	145 Gy	–	Yes (532%)	52	68	5-yr bRFS 79%
Chitmanee (UK)	2020	1	50	HDR	Ir-192	Focal	1 x 19 Gy	–	Yes (8%)	21 (1-53)	46	2-yr bRFS 63%, 3-yr bRFS 46%
Slevin (UK)	2020	1	43	HDR	Ir-192	Focal	1 x 19 Gy	–	Yes (74%)	26 (1-60)	79	3-yr bRFS 41.8%
van Son (Netherlands)	2020	1	50	HDR (MRI Guided ultra-focal)	Ir-192	Ultra-focal	1 x 19 Gy	–	Yes (12%)	31 (13-58)	48	2.5 yr bRFS 51%, mFS 75%, OS 98%
Kollmeier (USA)	2017	1	37	LDR	125-I (8%) or 103-Pd (92%)	Whole	125-144 Gy	–	Yes (46%)	31 (2-97)	65	3-yr bRFS 60.2%. 3-yr mFS 78.7%
			61	HDR	Ir-192	Whole	32 / 8 / 4 (n=58), 28 / 7 / 4 (n=1) and 22 / 11 / 2 (n=1)	30 hours	Yes (44%)			
Baumann (USA)	2017	1	33	HDR/LDR	103-Pd (LDR) and Ir-192 (HDR)	Whole	LDR (90-100 Gy) or HDR (30/6/5)	NR	Yes (100%)	61 (7-150)	67	7-yr RFS 67%
Henriquez (Spain)	2014	1	56	HDR/LDR	Ir-192/ 125-I	Whole	HDR: 50.5 / 5.25 / 1-4, LDR: 145 Gy	NR	Yes (26.8%)	48 (25-109)	NR	5-yr bRFS 77%, 5-yr OS 70%
Grado (USA)	1999	1	49	LDR	125-I (76%) or 103-Pd (24%)	Whole	80-180 Gy	–	Yes (16%)	41.7 (21.8-185.2)	34	3-yr bRFS 48%, 5-yr bRFS 34%. LC 98%
Koutrouvelis (USA)	2003	1	31	LDR	125-I (77%) or 103-Pd (23%)	Whole	100-144 Gy	–	No	30 (12-84)	87	3-yr bRFS 83.9%, 5-yr bRFS 41.9%
Nguyen (USA)	2007	1	25	LDR	125-I	Whole	137 Gy	–	No	47 (14-75)	72	4-yr bRFS 70%
HK Lee (USA)	2008	1	21	LDR	103-Pd	Whole	90 Gy	–	Yes (57%)	36	NA	5-yr bRFS 38%, 5-yr OS 81%
Aaronson (USA)	2009	1	24	LDR	125-I or 103-Pd	Whole	146 Gy	–	Yes (29%)	30 (13-65)	87.5	3-yr bRFS 89.5% 3-year CSS 96%
Burri (USA)	2010	1	37	LDR	103-Pd (97%) or 125-I (4%)	Whole	110-135 Gy	–	Yes (84%)	86 (2-156)	NA	5-yr bRFS 65%, 5-yr CSS 94%, 5-yr OS 96%
Moman (Netherlands)	2010	1	31	LDR	125-I	Whole	145 Gy	–	NA	108	19	1-yr bRFS 51%, 5-yr bRFS 20%, 5-yr CSS 74%, 5-yr OS 72%
Peters (Netherlands)	2014	1	20	LDR	125-I	Focal	144 Gy	–	NR	36 (10-45)	71	3-yr bRFS 71%
Vargas (USA)	2014	1	69	LDR	125-I	Whole	100 Gy	–	Yes (90%)	60 (7-164)	68.6	5-yr OS 64%, 5-yr mFS 90%
Peters (Netherlands)	2016	2	62	LDR (Whole Gland)	125-I	Whole	145 Gy	–	Yes (34%)	78 (5-139)	NR	Estimated 10-yr PCaSS 43%, 10-yr OS 34%
Crook (Canada)	2019	2	92	LDR	125-I (92%) or 103-Pd (8%)	Whole	120-140 Gy	–	NR	54	NR	NR
Smith (USA)	2020	2	108	LDR	125-I (1%) or 103-Pd (99%)	Whole	100 Gy	–	Yes (93.5%)	75 (1-228)	NR	5-yr bRFS 63%, 10-yr bRFS 52%
Schonle (Germany)	2020	1	82	PDR	Ir-192	Whole	60 / 30 / 2	4 weeks	Yes (43.9)	49 (12-129)	65.6	5-yr bRFS 65.6%, LC 86.6%

BT, brachytherapy; HDR, high dose rate; LDR, low dose rate; 125-I, Iodine-125; 103-Pd, Palladium-103; Ir-192, Iridium-192; Gy, Gray; ADT, androgen deprivation therapy; mo, months; BC, biochemical control; bRFS, biochemical recurrence free survival; mFS, metastasis free survival; RFS, relapse free survival; CSS, cancer specific survival; OS, overall survival.

Yamada (USA) study cohort included in further publication Kollmeier (USA) however specific treatment characteristics and toxicity were not covered in later paper.

**Table 7:** Salvage therapy details for EBRT studies.

First author/ country	Year	Single-centre (1) or Multi-centre (2)	Patients (n)	Treatment Technique	Delivery System	Dose (total dose (Gy)/ dose per fraction/ number of fractions)	Whole or Partial Gland/ Focal	Duration of treatment	Adjuvant ADT	Follow-up (mo) (range)	BC (%)	Oncologic outcomes
Fuller (USA)	2020	2	50	SBRT	Cyberknife	34 / 6.8 / 5	Whole	5 days	Yes (14%)	44 (3-110)	60	2-yr bRFS 76%, 5-yr bRFS 60%
Cuccia (Italy)	2020	1	24	SBRT	VMAT	30/06/05	Whole	5-12 days	Yes (16.7%)	21 (2-68)	54.9	1-yr bRFS 80%, 2-yr bRFS 54.9%, OS 100%
Matrone (Italy)	2020	1	44	SBRT	VMAT	35 / 5 / 7	Focal	7 days	Yes (27%)	25.4 (6.7-81.5)	59	1-yr bRFS 85.9%, 2-yr bRFS 58.3%, 2-yr LC 90.1%, 2-yr OS 100%
Caroli (Italy)	2020	1	38	SBRT	NR	18/ 6/ 3	Focal	3 days	NR	27 (4-35)	NR	bRFS 15 months
Bergamin (Australia)	2020	1	25	SBRT	VMAT	36 / 6 / 6 (72%) *vs* 38/ 6.3/ 6 (28%)	Focal	14 days	Yes (48%)	25 (13-46)	80	2-yr bRFS 80%
D'Agostino (Italy)	2019	1	23	SBRT	VMAT	25/ 5/ 5	Whole	5 days	Yes (43.5%)	33 (5-58)	34.8	2-yr bRFS 41.7%, 2-yr LC 61.1%, OS 100%
Pasquier (France)	2019	2	100	SBRT	Cyberknife (81%)/ VERO-IMRT, RapidArc	36 / 6 / 5	49% Whole *vs* 51% Partial	12 days	Yes (36%)	29.3 (4-91)	NR	bRFS 48 months, 3-yr bRFS 55%, 4-yr OS 94%
Scher (France)	2019	1	42	SBRT	Cyberknife	36 / 6 / 6	Focal	NR	Yes (19%)	21 (3-31)	94	median PFS 11 months, LC 100%
Jereczek-Fossa (Italy)	2018	1	64	SBRT	Cyberknife/ VERO-IMRT	30/ 6/ 5	Whole	5 days	Yes (25%)	26.1 (3.1-82.4)	64	2-yr bRFS 40%, LC 75%, OS 92%
Loi (Italy)	2018	1	50	SBRT	Cyberknife	30/ 6/ 5	NR	5 days	Yes (30%)	21.3 (6.1-49.2)	60	1-yr bRFS 80%, 1-yr mFS 92%
Leroy (France)	2017	1	23	SBRT	Cyberknife	36 / 6 / 6	83% Whole *vs* 17% Partial	14 days	Yes (61%)	22.6 (6-40)	54	2-yr bRFS 54%, OS 100%, 2-yr local dFS 76%

SBRT, Stereotactic body radiotherapy; VMAT, Volumetric modulated arc therapy; IMRT, Intensity-modulated radiation therapy; Gy, grey; ADT, androgen deprivation therapy; mo, months; BC, biochemical control; bRFS, biochemical recurrence free survival; mFS, metastasis free survival; RFS, relapse free survival; CSS, cancer specific survival; OS, overall survival; PFS, progression free survival.

Twenty-eight BT studies were included with a total of 1484 patients treated: 22 were retrospective and 6 were prospective. Four were multi-centre and 24 were single centre. Twelve BT studies used LDR only (16–27), 11 used HDR only (28–39), 4 used LDR or HDR (40–43) and 1 used PDR only (44). Twenty four studies used whole gland salvage treatments and 4 studies used focal salvage treatments (16, 34, 36, 38). The number of HDR-BT fractions ranged from 1 to 4 (median of 3 fractions) and the inter-fraction time interval ranged from 4 hours to 3 weeks. The median overall salvage treatment time was 21 days (range 1 to 63 days).

All EBRT studies (n=11) used an SBRT technique with a total of 483 patients treated. Of these studies (9, 45–54), 9 were retrospective and 2 were prospective (46, 47). Two were multi-centre and 9 were single centre. Four studies used Cyberknife delivery only and 7 studies included patients treated with Cyberknife or conventional linear accelerator SBRT techniques. Four studies used whole gland salvage only, 4 focal salvage only, 2 included both whole gland and focal treatments and one did not specify. Of the 11 studies, 8 were published between 2019 and 2020. The median total radiation dose prescribed was 34 Gy (range 34-38 Gy), over a median of 5 fractions (range 3-7). The median overall treatment time was 6 days (range 3-14 days).

The median number (range) of included patients for individual BT and SBRT studies was 44 (21-115) and 42 (23-100) respectively. The median age (range) at salvage treatment was 70 years (59-76) for BT studies and 74 years (64-78) for SBRT studies. The median PSA at primary treatment for the BT and SBRT studies were 10.9 ng/mL (range 7.4-26.4) and 10.3 ng/mL (range 8.7-13.0) respectively. The median PSA at salvage treatment for the BT and SBRT studies were 4.7 ng/mL (range 2.8-11.4) and 3.1 ng/mL (range 2.5-4.1) respectively. The median time from primary treatment to salvage therapy for the BT and SBRT studies were 67 months (range 30-101 months) and 86.5 months (range 60-100 months) respectively.

Seventeen studies (44%) used both multi-parametric magnetic resonance imaging (mpMRI) and positron emission tomography-computed tomography (PET-CT) for restaging prior to salvage treatment. Four studies (10%) used prostate-specific membrane antigen (PSMA) PET-CT and 13 studies (33%) used choline/fluciclovine PET-CT for re-staging. Eight studies (all BT) (21%) used computed tomography (CT) or isotope bone scintigraphy for restaging. Ten studies (26%) did not report the imaging modality used for restaging. Among the 28 BT studies, 24 included only patients with biopsy-proven local recurrence. Three of 11 SBRT studies included patients with histological confirmation of recurrence.

For BT studies, median follow up duration (range) was 47.5 months (13-108) compared with 25.4 months (21-44) for SBRT studies. The use of ADT with salvage therapy ranged from 8-100% in the BT study group and 14-61% in the SBRT group.

### Oncological Outcomes

For the LDR-BT studies, the median (range) 2-year and 5-year bRFS rates were 71% (48-89.5%) and 52.5% (20-79%). For the HDR-BT studies, the median (range) 2-year and 5-year bRFS rates were 74% (63-89%) and 51% (45-65%). For the SBRT studies, the median (range) 2-year bRFS for the SBRT group was 54.9% (40-80%). A 5-year estimate of bRFS following SBRT was only available for one study and was 60% (47). For focal gland BT, the median (range) 3-year bRFS was 63% (42-71%). For focal SBRT, the median (range) 3-year bRFS was 69% (58-80%). 3-year bRFS was presented as 2-year bRFS was not reported by the majority of these focal RT studies.

## Toxicity

A summary of clinician reported acute and late GU and GI toxicity data for each study is presented in **Table 8** (BT) and **Table 9** (SBRT).

**Table 8:** Toxicity details for BT studies.

First author (country)	Toxicity Scale	Acute GU toxicity		Acute GI toxicity		Late GU toxicity		Late GI toxicity		Erectile Dysfunction	PROMS
		Grade ≤ 2	Grade ≥ 3	Grade ≤ 2	Grade ≥ 3	Grade ≤ 2	Grade ≥ 3	Grade ≤ 2	Grade ≥ 3		
Kollmeier (USA)	CTCAE v4.0	96.0%	–	96.0%	–	82.0%	9.0%	91.0%	2.0%	NR	Yes (IPSS)
Baumann (USA)	CTCAE v4.0	82.0%	–	9.0%	–	42.0%	12.0%	3.0%	–	NR	Yes (IPSS)
Wojcieszek (Poland)	CTCAE v4.0	87.0%	1.0%	6.0%	–	72.0%	13.0%	6.0%	–	NR	NR
*Yamada (USA)	CTCAE v3.0	78.0%	–	NR	NR	86.0%	10.0%	57.0%	–	Yes	Yes (IPSS)
Peters (Netherlands)	CTCAE v4.0	100.0%	–	55.0%	–	40.0%	5.0%	35.0%	–	Yes (80%)	Yes (RAND-36, EORTC)
Vargas (USA)	NR	5.0%	8.7%	NR	NR	5.0%	8.7%	7.0%	3.0%	NR	NR
Chen (USA)	CTCAE v4.0	98.0%	2.0%	100.0%	–	98.0%	2.0%	100.0%	–	Yes (81%)	NR
Burri (USA)	CTCAE v3.0	35.0%	11.0%	5.0%	NR	35.0%	11.0%	NR	3.0%	Yes (75%)	NR
Moman (Netherlands)	CTCAE v3.0	87.0%	3.0%	55.0%	–	55.0%	19.0%	51.0%	6.0%	NR	NR
Aaronson (USA)	CTCAE v3.0	NR	NR	NR	3.0%	37.0%	4.0%	NR	4.0%	NR	Yes (IPSS, IIEF-5)
HK Lee (USA)	RTOG	29.0%	–	5.0%	–	29.0%	–	5.0%	–	NR	NR
Nguyen (USA)	RTOG	NR	NR	NR	NR	NR	20.0%	NR	20.0%	NR	NR
B Lee (USA)	CTCAE v3.0	86.0%	14.0%	14.0%	–	NR	5.0%	–	–	Yes (95%)	NR
Koutrouvelis (USA)	NR	13.0%	13.0%	13.0%	19.0%	13.0%	13.0%	13.0%	19.0%	NR	NR
Grado (USA)	NR	NR	NR	NR	NR	10.0%	20.0%	4.0%	2.0%	NR	NR
Slevin (UK)	CTCAE v4.0	91.0%	–	14.0%	–	65.0%	2.0%	14.0%	–	NR	NR
Lopez (Spain)	RTOG	33.0%	NR	NR	NR	NR	21.3%	NR	NR	NR	NR
	RTOG	33.0%	NR	NR	NR	NR	27.3%	NR	NR	NR	NR
Crook (Canada)	CTCAE v3.0	NR	14.0%	NR	14.0%	NR	7.0%	NR	4.0%	NR	Yes (IPSS)
Smith (USA)	CTCAE v5.0	NR	NR	NR	NR	NR	15.7%	NR	2.8%	Yes (80%)	Yes (IPSS, MSEFS)
Kukielka (Poland)	CTCAE v4.0	96.0%	–	12.0%	–	41.0%	–	–	–	NR	Yes (IPSS)
Schonle (Germany)	CTCAE v4.0	15.8%	6.1%	2.4%	–	15.8%	6.1%	2.4%	–	NR	NR
Chitmanee (UK)	NR	90.0%	–	32.0%	–	72.0%	10.0%	30.0%	–	Yes (86%)	Yes (IPSS)
Henriquez (Spain)	CTCAE v3.0	NR	NR	NR	NR	NR	23.0%	NR	4.0%	NR	NR
Peters (Netherlands)	CTCAE v4.03	NR	NR	NR	NR	NR	30.0%	NR	10.0%	NR	NR
Jiang (Germany)	CTCAE v4.0	100.0%	–	100.0%	–	90.9%	9.0%	100.0%	–	NR	Yes (IPSS)
van Son (Netherlands)	CTCAE v4.0	65.0%	–	37.0%	–	55.0%	2.0%	37.0%	–	Yes (100%)	Yes (IPSS, RAND-36)
Lacy (USA)	RTOG	9.6%	9.6%	9.6%	9.6%	9.6%	9.6%	9.6%	9.6%	Yes (45.5%)	Yes (IPSS)
Lyczek (Poland)	RTOG	29.6%	2.6%	7.9%	NR	7.0%	12.2%	1.7%	0.9%	NR	NR

- , 0% reported toxicity; NR, not reported; GU, genitourinary; GI, gastrointestinal; CTCAE, Common Terminology Criteria for Adverse Events; RTOG, Radiation Therapy Oncology Group; NR, not reported; PROMS, patient recorded outcome measures; IPSS, International prostate symptom score; RAND-36, RAND-36 Health Survey; EORTC, European Organisation for Research and Treatment of Cancer Quality of Life questionnaire; IIEF-5, International Index of Erectile Function questionnaire; MSEFS, Mount Sinai Erectile Function Score.

Yamada (USA) study cohort included in further publication Kollmeier (USA) however specific treatment characteristics and toxicity were not covered in later paper.

**Table 9:** Toxicity details for EBRT studies.

First author (country)	Toxicity Scale	Acute GU toxicity		Acute GI toxicity		Late GU toxicity		Late GI toxicity		Erectile Dysfunction	PROMS
		Grade ≤ 2	Grade ≥ 3	Grade ≤ 2	Grade ≥ 3	Grade ≤ 2	Grade ≥ 3	Grade ≤ 2	Grade ≥ 3		
Leroy (France)	CTCAE v4.0	78.2%	8.7%	17.4%	–	NR	NR	NR	NR	NR	NR
Fuller (USA)	CTCAE v3.0	2.2%	–	–	–	17.0%	8.0%	–	–	Yes (70%)	Yes (IPSS)
Jereczek-Fossa (Italy)	RTOG	25.0%	1.5%	9.5%	1.5%	37.0%	1.5%	7.5%	1.5%	NR	No
Loi (Italy)	CTCAE v3.0	20.0%	2.0%	8.0%	–	24.0%	2.0%	6.0%	–	NR	No
D'Agostino (Italy)	CTCAE v4.03	56.5%	4.4%	–	–	17.4%	4.4%	–	–	NR	No
Pasquier (France)	CTCAE v4.03	8.0%	1.0%	–	–	16.0%	1.0%	1.0%	–	NR	No
Scher (France)	CTCAE v4.03	64.0%	2.0%	7.0%	–	21.0%	2.0%	–	–	NR	No
Cuccia (Italy)	CTCAE v4.0	20.8%	–	–	–	12.5%	4.2%	4.2%	–	NR	No
Matrone (Italy)	RTOG	32.0%	–	8.0%	–	32.0%	4.0%	7.0%	–	NR	No
Caroli (Italy)	CTCAE v4.0	31.60%	–	31.60%	–	31.60%	–	31.60%	–	NR	No
Bergamin (Australia)	CTCAE v4.03	28.0%	–	8.0%	4.0%	32.0%	–	8.0%	–	NR	No

- , 0% reported toxicity; NR, not reported; GU, genitourinary; GI, gastrointestinal; CTCAE, Common Terminology Criteria for Adverse Events; RTOG, Radiation Therapy Oncology Group; NR, not reported; PROMS, patient recorded outcome measures; IPSS, International prostate symptom score.

In studies that only included LDR-BT, mean (range) grade 3 or higher toxicities were 7.4% (0-14%) (acute GU), 13.6% (0-30%) (late GU), 6.5% (0-19%) (acute GI) and 6.4% (0-20%) (late GI). In studies that only included HDR-BT, mean (range) grade 3 or higher toxicities were 2% (0-14%) (acute GU), 7.9% (0-21.3%) (late GU) and 0.1% (0-0.9%) (late GI). No grade 3 or higher acute GI toxicity was reported. For the SBRT group, mean (range) grade 3 or higher toxicities were 1.8% (0-8.7%) (acute GU), 2.7% (0-8%) (late GU), 0.5% (0-4%) (acute GI) and 0.2% (0-1.5%) (late GI).

For the focal gland BT group (n=4) (16, 34, 36, 38), the only grade 3 or higher toxicity reported was late GU toxicity – with a mean (range) of 4.8% (2-10%). For the focal gland SBRT group (n=4) (46, 51, 53, 54), mean (range) grade 3 or higher toxicities were 0.5% (0-2%) (acute GU), 1.5% (0-4%) (late GU) and 1% (0-4%) (acute GI). No late GI toxicity was reported.

Symptoms of erectile dysfunction were specifically reported by nine BT studies (32%) and one SBRT study (9%).

Thirteen studies (33.3%) (12 BT and one SBRT study) included PROMs, with the most common assessment tool used being the international prostate symptom score (IPSS).

## Discussion

This systematic review evaluated the most up-to-date evidence for salvage BT and SBRT and found that both treatment options provide good biochemical control with acceptable late GU/GI toxicity. However there is considerable heterogeneity between studies for numbers of patients, risk groups of included patients, evaluated treatments, reported endpoints, duration of follow up and methods of toxicity assessment (clinician-assessed versus PROMs). The quality of studies was low and meta-analysis was therefore not conducted due to the significant bias associated with these uncontrolled studies. This highlights the need for further high quality prospective and randomised studies to measure the efficacy and toxicity associated with salvage irradiation.

Consensus national and international recommendations for reirradiation are limited. The European Association of Urology (EAU) guidelines recommend salvage reirradiation using BT or SBRT for locally recurrent prostate cancer should only be undertaken in a clinical trial setting (55). American Society for Radiation Oncology (ASTRO) and National Comprehensive Cancer Network (NCCN) clinical practice guidelines does not comment on the use of reirradiation for prostate cancer however both the European Society of Radiation Oncology (ESTRO) and American Society of Brachytherapy (ABS) recommendations on prostate HDR-BT highlight the accumulating evidence for salvage HDR-BT in local recurrence as showing great promise (56–58).

There has been increasing interest in the use of salvage therapies for locally recurrent prostate cancer after primary radiation, although concerns have been raised regarding the potential for severe late toxicity (59). Both BT and SBRT, show durable outcomes in terms of biochemical control with reasonable reported toxicities in the majority of reviewed studies. However, inconsistencies in reporting and missing data preclude accurate comparison between these studies, which are mainly composed of case series. Longer term efficacy data and duration of follow up was available for more BT studies than SBRT but, at short term follow-up, the clinician-reported toxicity following salvage SBRT appear to be infrequent (60).

Two previous meta-analyses which compared salvage therapies in recurrent prostate cancer have been conducted, which included radical prostatectomy, cryotherapy and HIFU in addition to BT and EBRT (10, 61). The meta-analysis by Valle et al. (61) reported that recurrence free survival and toxicity rates were best for salvage radiotherapeutic modalities compared to other salvage treatments, and BT appeared to offer the best balance between toxicity and efficacy. For example, the estimated recurrence free survival at 2 years for BT was 77-79% compared to 52-72% for cryotherapy, HIFU and salvage radical prostatectomy. In addition, lower grade 3 or higher GU toxicity was observed (5-10% versus 20% for BT compared with other salvage therapies) (61). The quality of the evidence was not assessed and sub-group and sensitivity analyses to explore potential impact of clinical heterogeneity were also not specified in this meta-analysis (61). In addition, it was unclear how many studies were excluded from the meta-analysis due to incompatible definitions, outcome measures and follow-up periods. Interestingly, the 2-year bRFS of SBRT (54.9%) appeared to be lower than both LDR- and HDR-BT (71% and 74% respectively). A formal comparison between these modalities is limited by confounding factors, although these data raise an interesting question as to whether salvage SBRT could be inferior to BT in terms of biochemical control.

Comparing the SBRT studies to BT remains challenging in view of the heterogeneous populations and shorter follow-up available for SBRT with only one study providing 5-yr bRFS data (although in this study, comparable to outcomes from salvage BT were reported) (47). No prospective randomised studies exist which compare BT and SBRT as salvage therapies for locally recurrent prostate cancer and this is ultimately what is required.

There may be dosimetric advantages with the use of BT compared with SBRT. A previous planning study in the primary disease setting concluded that HDR-BT was able to achieve higher intraprostatic doses and greater sparing of the rectum than SBRT (62). It is possible that developments in SBRT planning and delivery might lead to improved outcomes. For example, the superior soft tissue visualisation and functional imaging capabilities of MR guided SBRT might permit better delineation of tumour, greater accuracy of treatment delivery and offer opportunities for dose escalation (63). Whether this would translate into a clinical benefit at this point remains uncertain. There remains considerable interest in salvage SBRT as evidenced by the fact that 8 of 11 SBRT studies were published in the last two years.

Based on the studies evaluated in this review, salvage LDR-BT appeared to have the potential for higher grade 3+ toxicity compared to HDR-BT (19, 24). In a study which used PROMs, LDR-BT had a higher peak change in IPSS in the early post-implant period and a higher peak urinary symptom flare at 12 months compared with HDR-BT, although the majority of these scores returned to baseline 2-3 years post-treatment (40). There have been no prospective studies comparing these techniques in the reirradiation setting. In the primary treatment setting however prospective and randomised studies have shown HDR-BT to have better quality of life scores compared to LDR-BT in the acute post-treatment phase, particularly in the urinary health domain, which suggests that HDR was better tolerated (64, 65). Similarly, evidence from registries and randomised trials of LDR/EBRT combination therapy and HDR/EBRT combination therapy in the primary disease setting suggest that LDR/EBRT might be associated with higher incidence of significant late GU toxicity although no direct comparison has been performed between the two treatments (66–68).

Based on the available data, grade 3 or higher GU and GI toxicity with SBRT was rare, although follow-up beyond 2 years is limited (9, 45–54). SBRT has the potential to limit the risk of severe late GU/GI toxicity compared with less conformal EBRT techniques (69). Careful patient selection remains vital, especially for those at greater risk of excess toxicity following salvage therapy. In a recent observational series of salvage SBRT, grade 3+ GU toxicity was disproportionately observed in patients treated with BT or radical prostatectomy plus salvage RT in the primary disease setting (47). Furthermore, the use of focal salvage techniques with BT and SBRT appear to have lower toxicity rates and comparable bRFS rates however this is limited to a number of uncontrolled, single-arm case series (16, 34, 36, 38, 46, 51, 53, 54).

Appropriate patient selection for salvage RT treatments is vital. The European Society for Radiotherapy and Oncology Advisory Committee on Radiation Oncology Practice (ESTRO ACROP) recently conducted a Delphi consensus of expert opinion on patient selection criteria for salvage RT (70). Selection criteria with high levels of agreement (>80%) included Eastern Cooperative Oncology Group performance status of 0-1, satisfactory urinary flow with a known IPSS prior to salvage and use of PET-CT to exclude metastatic disease and MRI to define the target. Agreement was reached that concomitant ADT with salvage RT was unnecessary and that previous ADT use was not a contraindication to salvage RT. It was also recommended that the primary RT dose should be taken into account when considering salvage SBRT. In terms of time duration between primary RT and salvage RT, although consensus was not achieved a minimum interval of 2 years reached major agreement (defined as 65-80% agreement).

The impact on quality of life has not been well assessed in the salvage radiotherapy setting with only a third of studies in this systematic review including PROMs. Only one of 11 SBRT (9.1%) studies included PROMs. Without this information, it is likely that reported rates of toxicity are underestimated (71). Assessment of residual toxicity following primary treatment using validated PROM instruments such as Expanded Prostate Cancer Index (EPIC) could be an important tool for identification of patients at risk of significant toxicity from salvage therapies. Integration of longitudinal PROM assessment into clinical trials is important to ascertain the time-dependent nature of toxicity onset/resolution after treatment (71, 72).

The role of ADT with salvage BT/EBRT remains unclear and no consensus could be reached during a previous Delphi consensus (73). The use of ADT with salvage radiation therapy in the evaluated studies was highly variable (8-100%) and reporting of ADT duration was incomplete (16, 32, 36, 41, 74). Several BT studies did not report ADT usage or did not use neoadjuvant ADT (24, 25, 33). Salvage therapies may delay the need for ADT, with up to 69% patients remaining free of ADT at 5 years following salvage SBRT (47). Some authors view salvage BT/SBRT as ADT sparing, which might have the potential to improve quality of life (75).

A recent study found that only 15% of relapses following salvage BT were solely in the prostate (36), suggesting most are likely to be systemic failure therefore accurate and consistent whole body imaging staging is imperative. The optimal combination of re-staging imaging following biochemical failure after primary treatment, and the most clinically relevant PSA level at which to trigger such imaging, remains uncertain (76). Despite the poor accuracy of CT and isotope bone scintigraphy, 21% of studies in this systematic review used these modalities for restaging and patient selection. It is possible that some patients in these studies could have had undiagnosed metastatic disease, and this could be responsible for some subsequent biochemical failures (75, 77). Less than half of studies used mpMRI and PET-CT for re-staging prior to salvage therapy. mpMRI has the potential to be particularly useful for detecting local recurrence following previous prostate radiotherapy, although studies evaluating its accuracy are limited (78). The use of novel imaging modalities such as Gallium-68 [^68^Ga] or Fluorine-18 [^18^F] labelled PSMA PET-CT, may allow detection of local recurrence at lower PSA levels. While this could lead to a change in management for patients identified with recurrent disease, it was only used in 10% of the studies in this systematic review (79). ^68^Ga-PSMA PET-CT has been shown to demonstrate recurrences at prostate-specific antigen (PSA) levels below the Phoenix definition of biochemical failure and it allows for both local staging and exclusion of distant metastatic disease in patients with biochemical failure (80). The recent proPSMA randomised study reported that PSMA PET-CT had a greater accuracy compared to conventional imaging with CT and bone scan in the primary setting (92% vs 65%) (81). PSMA PET-CT also has superior performance characteristics for the detection of distant metastasis in the setting of biochemical failure compared to other PET tracers (82). Nevertheless, the clinical significance of detecting and treating small volume local recurrence at low PSA levels remains uncertain and may risk additional toxicity. Prospective randomised trials comparing BT and SBRT for salvage treatment of locally recurrent prostate cancer are required to determine the efficacy/toxicity of these interventions

## Summary of Main Findings

- For the LDR-BT studies, the median (range) 2-year and 5-year bRFS rates were 71% (48-89.5%) and 52.5% (20-79%).
- For the HDR-BT studies, the median (range) 2-year and 5-year bRFS rates were 74% (63-89%) and 51% (45-65%).
- For the SBRT studies, the median (range) 2-year bRFS for the SBRT group was 54.9% (40-80%).
- For LDR-BT, mean (range) grade 3 or higher toxicities were 7.4% (0-14%) (acute GU), 13.6% (0-30%) (late GU), 6.5% (0-19%) (acute GI) and 6.4% (0-20%) (late GI).
- For HDR-BT, mean (range) grade 3 or higher toxicities were 2% (0-14%) (acute GU), 7.9% (0-21.3%) (late GU) and 0.1% (0-0.9%) (late GI). No grade 3 or higher acute GI toxicity was reported.
- For SBRT, mean (range) grade 3 or higher toxicities were 1.8% (0-8.7%) (acute GU), 2.7% (0-8%) (late GU), 0.5% (0-4%) (acute GI) and 0.2% (0-1.5%) (late GI).
- Only thirteen studies (33.3%) included PROMs, with the most common assessment tool used being IPSS.

## Limitations

The overall quality of evaluated evidence was low. A meta-analysis was not conducted to quantitatively compare the studies as the majority of these were non-comparative retrospective case series with differences in baseline patient demographics, primary and/or salvage treatments, reported endpoints reported and use of ADT. This limits the conclusions that can be drawn about the effectiveness/toxicity of salvage BT/SBRT. High-quality data from prospective trials are still needed to validate the toxicity and long-term clinical outcomes associated with the salvage treatment of recurrent prostate cancer using BT or EBRT, following previous RT.

## Conclusions

Salvage reirradiation of radiorecurrent prostate cancer using HDR-BT or SBRT provides similar biochemical control and acceptable late toxicity. Salvage LDR-BT is associated with higher late GU/GI toxicity. Challenges exist in comparing BT and SBRT from the current literature due to inconsistencies in reporting and missing data. Prospective randomised trials comparing BT and SBRT and assessing PROMs as well as cancer control outcomes in this setting are needed.

## Data Availability Statement

The original contributions presented in the study are included in the article/**Supplementary Material**. Further inquiries can be directed to the corresponding author.

## Author Contributions

All authors contributed to the article and approved the submitted version. JZ and FS did the literature search, assembly of data and data analysis. JZ, FS and AH: data interpretation to ensure relevance of findings.

## Funding

JZ is a Clinical Research Fellow supported by a Cancer Research UK Award (Leeds-Manchester Stella Erdheim Clinical PhD Fellowship – Grant Reference Number 95653117/95653118). FS is a Clinical Research Fellow supported by a Cancer Research UK Centres Network Accelerator Award to the ART-NET Consortium (Grant number A21993). AS receives salary support for academic work from Cancer Research UK *via* the Radiation Research Centre of Excellence at the University of Leeds (Grant Reference Number C19942/A2882). AC and PH are supported by NIHR Manchester Biomedical Research Centre. AH is supported by grants from Cancer Research UK (award number 108036), National Institute for Health Research (NIHR) (award number 111218), Medical Research Council (MRC) (award number 107154) and Sir John Fisher Foundation.

## Conflict of Interest

AH served as a guest editor for Frontiers in Oncology.

The remaining authors declare that the research was conducted in the absence of any commercial or financial relationships that could be construed as a potential conflict of interest.

The reviewer NJ declared a past collaboration with the authors AC, PH to the handling editor.

## Publisher’s Note

All claims expressed in this article are solely those of the authors and do not necessarily represent those of their affiliated organizations, or those of the publisher, the editors and the reviewers. Any product that may be evaluated in this article, or claim that may be made by its manufacturer, is not guaranteed or endorsed by the publisher.
