# Supplementary material for: Salvage Reirradiation Options for Locally Recurrent Prostate Cancer: A Systematic Review
Source: Front Oncol. 2021 Sep 9;11:681448. doi: 10.3389/fonc.2021.681448 (PMC8459721; doi:10.3389/fonc.2021.681448)
Supplement: Supplementary file 1 [file DataSheet_1.docx]

**EMBASE and MEDLINE Search Strategies:**

From inception of databases until December 14th 2020.

**EMBASE**

1. prostat*.mp.

2. (external beam* or ebrt or brachytherapy or hypofractionated or stereotactic or sabr or sbrt or re-irradiation or reirradiation or retreatment or radiosurgery).mp.

3. (repeat or recurr* or relapse or salvage).mp.

4. 1 and 2 and 3

Exclude Medline Journals

**MEDLINE**

prostat*[tw]

AND

(external beam*[tw] OR ebrt[tw] OR brachytherapy[tw] OR hypofractionated[tw] OR stereotactic[tw] OR sabr[tw] OR sbrt[tw] OR re-irradiation[tw] OR reirradiation[tw] OR radiosurgery[mh])

AND

(repeat[tw] OR recurr*[tw] OR relapse[tw] OR salvage[tw])
