# Supplementary material for: Salvage Reirradiation Options for Locally Recurrent Prostate Cancer: A Systematic Review
Source: Front Oncol. 2021 Sep 9;11:681448. doi: 10.3389/fonc.2021.681448 (PMC8459721; doi:10.3389/fonc.2021.681448)
Supplement: Supplementary file 2 [file Table_1.docx]

Supplementary Table 1 - Modified Delphi Checklist for quality assessment results for each included study

|  |  |  | **Modified Delphi Checklist (18-items)** | | | | | | | | | | | | | | | | | |
| --- | --- | --- | --- | --- | --- | --- | --- | --- | --- | --- | --- | --- | --- | --- | --- | --- | --- | --- | --- | --- |
| **Study** | **Year** | **Treatment** | **1** | **2** | **3** | **4** | **5** | **6** | **7** | **8** | **9** | **10** | **11** | **12** | **13** | **14** | **15** | **16** | **17** | **18** |
| Slevin | 2020 | BT | Y | Y | N | N | Y | Y | Y | Y | Y | Y | Y | Y | Y | N | N | Y | Y | Y |
| Smith | 2020 | BT | Y | Y | Y | Y | Y | Y | Y | Y | Y | Y | Y | Y | Y | Y | N | Y | Y | Y |
| Schonle | 2020 | BT | Y | Y | N | Y | N | Y | Y | Y | Y | Y | Y | Y | Y | Y | N | Y | Y | N |
| Chitmanee | 2020 | BT | Y | Y | N | Y | Y | Y | Y | Y | Y | Y | Y | Y | Y | N | N | Y | Y | Y |
| Van Son | 2020 | BT | Y | Y | N | Y | Y | Y | Y | Y | Y | Y | Y | Y | Y | N | Y | Y | Y | Y |
| Lopez | 2019 | BT | Y | Y | Y | Y | Y | Y | Y | Y | Y | Y | Y | Y | Y | N | Y | Y | Y | Y |
| Crook | 2019 | BT | Y | Y | Y | Y | Y | Y | Y | Y | Y | Y | Y | Y | Y | Y | Y | Y | Y | Y |
| Kollmeier | 2017 | BT | Y | Y | N | N | Y | Y | Y | Y | Y | Y | Y | Y | Y | N | Y | Y | Y | N |
| Baumann | 2017 | BT | Y | Y | Y | Y | Y | Y | Y | Y | Y | Y | Y | Y | Y | N | Y | Y | Y | N |
| Wojcieszek | 2016 | BT | Y | Y | N | Y | Y | Y | Y | Y | Y | Y | Y | Y | Y | Y | Y | Y | Y | Y |
| Peters | 2016 | BT | Y | Y | Y | Y | N | Y | Y | Y | Y | Y | Y | Y | Y | Y | Y | Y | Y | Y |
| Jiang | 2016 | BT | Y | Y | N | Y | N | Y | Y | Y | Y | Y | Y | Y | Y | Y | N | Y | Y | N |
| Lacy | 2016 | BT | Y | Y | N | N | N | N | Y | Y | Y | Y | Y | Y | Y | Y | N | Y | Y | N |
| Yamada | 2014 | BT | Y | Y | N | Y | Y | Y | Y | Y | Y | Y | Y | Y | Y | Y | Y | Y | Y | Y |
| Peters | 2014 | BT | Y | Y | N | Y | Y | Y | Y | Y | Y | Y | Y | Y | Y | Y | N | Y | Y | Y |
| Vargas | 2014 | BT | Y | Y | N | Y | Y | Y | Y | Y | Y | Y | N | Y | Y | Y | N | Y | Y | Y |
| Kukielka | 2014 | BT | Y | Y | N | Y | Y | Y | Y | Y | Y | Y | Y | Y | Y | N | N | Y | Y | Y |
| Henriquez | 2014 | BT | Y | Y | Y | Y | Y | Y | Y | Y | Y | Y | Y | Y | Y | N | N | Y | Y | N |
| Chen | 2013 | BT | Y | Y | N | Y | Y | Y | Y | Y | Y | Y | Y | Y | Y | N | Y | Y | Y | Y |
| Burri | 2010 | BT | Y | Y | N | Y | N | Y | Y | Y | Y | Y | Y | Y | Y | Y | N | Y | Y | Y |
| Moman | 2010 | BT | Y | Y | N | Y | N | Y | Y | Y | Y | Y | Y | Y | Y | Y | N | Y | Y | Y |
| Aaronson | 2009 | BT | Y | Y | N | Y | Y | Y | Y | Y | Y | Y | Y | Y | Y | Y | N | Y | Y | Y |
| Lyczek | 2009 | BT | Y | Y | N | N | N | Y | N | N | N | N | N | Y | N | N | N | Y | Y | N |
| Lee | 2008 | BT | Y | Y | N | Y | Y | Y | Y | N | Y | Y | N | Y | Y | N | N | Y | Y | N |
| Nguyen | 2007 | BT | Y | Y | N | Y | Y | Y | Y | Y | Y | Y | Y | Y | Y | Y | N | Y | Y | Y |
| Lee | 2007 | BT | Y | Y | N | N | Y | Y | Y | N | Y | Y | Y | Y | Y | N | N | Y | Y | Y |
| Koutrouvelis | 2003 | BT | N | Y | N | Y | N | Y | Y | Y | Y | Y | N | N | Y | Y | N | N | N | N |
| Grado | 1999 | BT | Y | Y | N | N | Y | N | Y | Y | Y | Y | Y | Y | Y | N | Y | Y | Y | N |
| Fuller | 2020 | EBRT | Y | Y | Y | Y | Y | Y | Y | Y | Y | Y | Y | Y | Y | Y | N | Y | Y | N |
| Cuccia | 2020 | EBRT | Y | Y | N | Y | N | Y | Y | Y | Y | Y | Y | Y | Y | N | N | Y | Y | N |
| Matrone | 2020 | EBRT | Y | Y | N | Y | Y | N | Y | Y | Y | Y | Y | Y | Y | N | Y | Y | Y | Y |
| Caroli | 2020 | EBRT | Y | Y | N | Y | Y | N | N | N | Y | Y | Y | Y | Y | N | Y | Y | Y | Y |
| Bergamin | 2020 | EBRT | Y | Y | N | Y | Y | Y | Y | Y | Y | Y | Y | Y | Y | Y | Y | Y | Y | Y |
| D'Agostino | 2019 | EBRT | Y | Y | N | Y | N | Y | Y | Y | Y | Y | Y | Y | Y | Y | N | Y | Y | N |
| Pasquier | 2019 | EBRT | Y | Y | Y | Y | N | Y | Y | Y | Y | Y | Y | Y | Y | Y | Y | Y | Y | N |
| Scher | 2019 | EBRT | Y | Y | N | Y | N | Y | Y | Y | Y | Y | Y | Y | Y | Y | N | Y | Y | N |
| Jereczek-Fossa | 2018 | EBRT | Y | Y | N | Y | Y | Y | Y | Y | Y | Y | Y | Y | Y | Y | N | Y | Y | Y |
| Loi | 2018 | EBRT | Y | Y | N | Y | Y | Y | Y | Y | Y | Y | Y | Y | Y | Y | N | Y | Y | N |
| Leroy | 2017 | EBRT | Y | Y | N | Y | N | Y | Y | Y | Y | Y | Y | Y | Y | Y | Y | Y | Y | Y |
